# MALAT1 as a Regulator of the Androgen-Dependent Choline Kinase A Gene in the Metabolic Rewiring of Prostate Cancer

**DOI:** 10.3390/cancers14122902

**Published:** 2022-06-12

**Authors:** Sara De Martino, Egidio Iorio, Chiara Cencioni, Aurora Aiello, Francesco Spallotta, Mattea Chirico, Maria Elena Pisanu, Claudio Grassi, Alfredo Pontecorvi, Carlo Gaetano, Simona Nanni, Antonella Farsetti

**Affiliations:** 1 Department of Translational Medicine and Surgery, Faculty of Medicine and Surgery, Università Cattolica del Sacro Cuore, 00168 Rome, Italy; sara.demartino@unicatt.it; 2 Istituto Superiore di Sanità, 00161 Rome, Italy; egidio.iorio@iss.it (E.I.); mattea.chirico@iss.it (M.C.); mariaelena.pisanu@iss.it (M.E.P.); 3 National Research Council (CNR)-IASI, 00185 Rome, Italy; chiara.cencioni@cnr.it (C.C.); aurora.aiello@cnr.it (A.A.); francesco.spallotta@cnr.it (F.S.); 4 Fondazione Policlinico Universitario A. Gemelli IRCCS, Università Cattolica del Sacro Cuore, 00168 Rome, Italy; claudio.grassi@unicatt.it (C.G.); alfredo.pontecorvi@unicatt.it (A.P.); 5 Istituti Clinici Scientifici Maugeri, 27100 Pavia, Italy; carlo.gaetano@icsmaugeri.it

**Keywords:** choline kinase A, prostate cancer, MALAT1, metabolic rewiring, phospholipid metabolism

## Abstract

**Simple Summary:**

Despite the rapid advance in cancer therapies, treatment-resistant relapse remains a significant challenge in cancer treatment. Acquired resistance arises during or after treatment administration, and is usually the main contributor to relapse. For example, prostate cancer, the most frequent type of cancer in the elderly male population, frequently develops into aggressive forms resistant to chemical and hormonal therapies. In this condition, the so-called “cholinic phenotype” that is characterized by the overexpression of choline kinase alpha (CHKA) and increased phosphocholine levels leads to aberrant lipid metabolism. Our work demonstrates that CHKA, which is necessary for membrane phospholipid synthesis, is a target of the long non-coding RNA MALAT1. This study helps to further decipher how MALAT1 affects the regulation of crucial phospholipid/sphingolipid metabolic enzymes, as well as how the androgen receptor pathway is involved in MALAT1-dependent transcriptional regulation.

**Abstract:**

Background. Choline kinase alpha (CHKA), an essential gene in phospholipid metabolism, is among the modulated MALAT1-targeted transcripts in advanced and metastatic prostate cancer (PCa). Methods. We analyzed CHKA mRNA by qPCR upon MALAT1 targeting in PCa cells, which is characterized by high dose-responsiveness to the androgen receptor (AR) and its variants. Metabolome analysis of MALAT1-depleted cells was performed by quantitative High-resolution 1 H-Nuclear Magnetic Resonance (NMR) spectroscopy. In addition, CHKA genomic regions were evaluated by chromatin immunoprecipitation (ChIP) in order to assess MALAT1-dependent histone-tail modifications and AR recruitment. Results. In MALAT1-depleted cells, the decrease of CHKA gene expression was associated with reduced total choline-containing metabolites compared to controls, particularly phosphocholine (PCho). Upon MALAT1 targeting a significant increase in repressive histone modifications was observed at the CHKA intron-2, encompassing relevant AR binding sites. Combining of MALAT1 targeting with androgen treatment prevented MALAT1-dependent CHKA silencing in androgen-responsive (LNCaP) cells, while it did not in hormone-refractory cells (22RV1 cells). Moreover, AR nuclear translocation and its activation were detected by confocal microscopy analysis and ChIP upon MALAT1 targeting or androgen treatment. Conclusions. These findings support the role of MALAT1 as a CHKA activator through putative association with the liganded or unliganded AR, unveiling its targeting as a therapeutic option from a metabolic rewiring perspective.

## 1. Introduction

The average age in Western countries is increasing rapidly, and with it the incidence of aging-associated cancers [1]. Finding effective therapeutic strategies targeting the most resistant forms of aging-associated tumors is a recent unmet need. Prostate cancer (PCa) is the most frequent type of cancer in the elderly male population [2,3]. It frequently develops into aggressive forms resistant to chemical and hormonal therapies. In this condition, the so-called “cholinic phenotype”, which is characterized by the overexpression of choline kinase alpha (CHKA) and increased phosphocholine (PCho) levels, supports aberrant lipid metabolic pathways typical of different cancers, including PCa. Choline kinase (CHK) has already been identified as a promising target for controlling castration-resistant prostate cancer (CRPC) through modulation of androgen receptor (AR) signaling [4,5]. 

CHK exists in three isoforms, CHKA-1, CHKA-2, and CHKB. These isoforms are encoded by two separate genes, *CHKA* and *CHKB*, and are active in homodimeric, heterodimeric, and oligomeric forms [6]. Increased expression and enzymatic activity of CHKA-1/2 have been identified in human malignancies, including breast, lung, colorectal, bladder, prostate, ovarian, endometrial carcinomas, osteosarcoma, and T-cell lymphoma [7,8,9]. Therefore, the upregulation of CHK activity in cancer probably results from an increase in CHKA expression, which would lead to a higher proportion of CHKA-]–CHKA dimers exerting a higher CHK activity level than CHKA–CHKB heterodimers or CHKB–CHKB homodimers [10]. 

CHK catalyzes the formation of PCho, the committed step in phosphatidylcholine biosynthesis. Phosphatidylcholine (PC) is the major phospholipid in eukaryotic membranes with different essential functions, including (i) cholesterol transport support through the organism; (ii) substrate for the production of second messengers; and (iii) cofactor for the activity of several membrane-related enzymes [11]. Notably, CHK-derived PC is necessary for mitogenesis-related pathways, especially in the proliferation of human mammary epithelial cells [12]. In this light, alterations in choline metabolism, typical in many cancer cell types, are thought to reflect the increased demands of proliferating cancer cells [10]. 

Furthermore, CHK plays a vital role in producing Sphingomyelin, another essential membrane phospholipid crucial for cell growth [13]. The enhanced lipid de novo synthesis during tumorigenesis supports the rapid proliferation of cancer cells, cell signaling, and tumor survival [10,14]. 

In PCa, CHKA acts as an AR co-chaperone, supporting its signaling [5]. The working hypothesis is that a reduction in CHKA function may negatively influence AR activity and PCa growth. Blockade of this enzyme induces cells to activate a different route for phospholipid production, which causes a toxic effect and eventually leads to cell destruction. CHKA plays a significant role in cellular proliferation, apoptosis evasion, cell motility, and metastasis. Consistently, shRNA-mediated in vivo depletion of CHKA decreases the growth of prostate tumor xenografts [5]. Very efficient CHKA inhibitors have been developed and considered in clinical trials, however, they have manifested elevated toxicity, as reported in [7,15,16,17]. Hence, alternative strategies should be considered.

We previously reported that the long noncoding RNA MALAT1, which is involved in cancer progression and metastasization, is efficiently targeted by specific gapmers in Pca cell lines and an ex vivo model of organotypic slice cultures (OSCs) derived from Pca surgery tissue explants [18]. Of interest, MALAT1 depletion determined a profound reprogramming of cancer cells and tumor tissue metabolism, causing a switch toward a more glycolytic phenotype, which is unusual for PCa and does not efficiently support tumor growth [18]. In the present work, we observe that MALAT1 targeting broadly hurts the cholinic phenotype via the downregulation of CHKA and PCho synthesis, with essential consequences on tumor cell proliferation.

## 2. Materials and Methods 

Antibodies: Androgen Receptor (1:1000, Merk Millipore, Burlington, MA, USA, #06680); CHKA (1:500, Sigma-Aldrich, St. Louis, MO, USA, HPA-024153); β-actin (1:8000, Sigma, #1305556); CERK (1:1000, Abcam, Cambridge, UK, #155061); GAPDH (as described in [18]; Bcl2 (1:1000, Biotechnology, Dallas TX, USA, M0887); Fibrillarin (1:1000, ThermoFisher, Waltham, MA, USA, MA3-16771); Tubulin (1:5000, Cell Signaling, Danvers, MA, USA, #2146).

Cell cultures, treatments, and MALAT1 silencing: LNCaP, PC3, PC3-AR, and 22RV1 cells were grown in RPMI medium (1640 Corning, New York, USA, #10-040-CV), and DU145 cells were grown in MEM medium (Corning, #15-010-CVR); C27IM and HUVEC cells were grown as in [19]. All media were supplemented with 10% FBS (Gibco, Invitrogen, Carlsbad, CA, USA, #10270106), 1% glutamine (Corning #25005-CI), 1% penicillin and streptomycin (Corning #30002-CI), 1% HEPES (Corning #25060-CI) for LNCaP and 22RV1 cells, and 1% sodium pyruvate (Corning #25000-CIR) and 1% glucose for 22RV1 cells. Cells were incubated at 37 °C with 5% CO_2_. At least 72 h before use, LNCaP and 22RV1 cells were switched to a medium with hormone-deprived serum as in [19] and treated with 5 α-Dihydrotestosterone (DHT) (NMID, Sydney, NSW, AU, #680) with the concentrations and times indicated in the figure legends. BMR Genomics authenticated the genetic identity of the PC3, DU145, and C27IM cell lines as previously described in [18]. LNCaP cells were obtained from the American Type Culture Collection. PC3AR and 22RV1 cells were kindly provided by Prof. Aria Baniahmad (Institute for Human Genetics and Anthropology, Friedrich-Schiller-University, Jena, Germany) and Prof. Claudio Sette (Department of Neuroscience, Catholic University, Rome, Italy), respectively. MALAT1 silencing was obtained using specific MALAT1 gapmers. LacZgapmers were used as a negative control as described in [18,19]. 

RNA extraction and real-time qPCR. RNA extraction and real-time PCR were performed on QuantStudio 5 Real-Time PCR System (Applied Biosystems, Foster City, CA, USA) using SYBR Green quantification as in [18]. Quantification was performed using 2^−ΔΔCt^ as in [18]; data are expressed as fold change. In addition, the following primers (forward and reverse, respectively) were used:

hCHKA 5′-GGTCACTTGGGCCAAAACTC-3’ and 5′-GCCGGCTCGGGATGA-3′;
hPLCG1 5′-GCTTCTATGTAGAGGCAAACCCTATG-3′ and 5′-CCCTCTGGGCCTTGTAGTCA-3′
hSMPD1 5′-TGCCCAATCTGCAAAGGTCTA-3′ and 5′-GCCACATTGGGTTCCTTCTTC-3′
hSGMS1 5′-AGCATGATTCAGGCACACCAT-3′ and 5′-TCATGTTTCCCAACCAGACACT-3′
hCERK 5′-TGGCACCACTGTTCACCTTA-3′ and 5′-CTCCTTGGCCTGATTAGCAT-3′

Primers to MALAT1, PSA, GAPDH, P0, and β-actin were as in [19,20].

Protein extraction and western blot: Total and Nuclear/Cytoplasmatic protein extract was performed as in [19] and [18], respectively. Western blot assay was performed using 25 µg of protein extract and proteins solved by SDS-PAGE. Protein signals were revealed with ECL Prime (Amersham, GE Healthcare, Boston, MA, USA) and detected by UVITEC (Eppendorf S.r.l., Hamburg, Germany). Densitometric analysis was performed with NIH Image J 1.8 software (National Institutes of Health, Bethesda, MD, USA) and specific values were normalized to loading control (βActin, GAPDH, tubulin, and fibrillarin, as indicated). All original western blots can be found in File S1.

Chromatin Immunoprecipitation (ChIP). ChIP was performed in PC3 (N = 2) and LNCaP (N = 2) cells as in [19,20]. Briefly, DNA fragments were recovered and analyzed by qPCR on QuantStudio 5 Real-Time PCR System (Applied Biosystems) using SYBR Master Mix (Applied Biosystems, Foster City, CA, USA) with the evaluation of dissociation curves. DNA Input serial dilutions were used as standard curves and data normalized to corresponding inputs were expressed as relative enrichment. Immunoprecipitations were performed using specific antibodies to H3K4me3 (Active Motif, Carlsbad, CA, USA, #39160), H3Ac (Millipore, #06599), H3K9me3 (Active Motif, #39162), EZH2 (D2C9, Cell Signaling, #5246), H3K27me3 (Active Motif, #39157), and AR (441, Santa Cruz Biotechnology, Dallas, TX, USA, #7305, C-19, Santa Cruz #815). No antibody or IgG (Bethyl, Montgomery, TX, USA, #P120-101) were used as negative controls. Primer sequences (forward and reverse, respectively) are listed below:

CHKAprom (−3500) 5′-GGAAAAGGTTTGGTAATTGGAACA-3′ and 5′-CTGTGCACAAGTAGACGAGTTTGA-3′
CHKAprom (−1500) 5′-CGCCACAGCAGCCTTACAA-3′ and 5′-CCCAAAGTGCTGGGATTACAG-3′
CHKAprom (Intron 2, 37,500) 5′-CAGGACTTAGGGAGCCTGAACA-3′ and 5′-GGCAAGATGGACTTCTGCAATAT-3′.
hCHKA (Intron 2, 27,500) 5′-GATGGGAGTAATGGAGGGTTCTG-3′ and 5′-CGTTAGTGACATGTGGCTGATGA-3′
CERKprom (TSS) 5′-ACAAGACGGACTGTGGATGGA-3′ and 5′-CATCTGTTCTTGGAGTAAACTGCAA-3′
CERKprom (−8600) 5′-GCCACTCTGTTCTGCGATCAC-3′ and 5′-CTGTTGGAGCCTCCGTTTTC-3′
CERKprom (Intron, 46,500) 5′-CTTGGGAGACGGGTCTTCTG-3′ and 5′-GGTTGGTGTGCCTGATGAGA-3′

Putative AR-binding sites on the CHKA gene (Intron 2, 37,500 bp from TSS; −1500 bp from TSS) were identified using both MatInspector (Genomatix, Munich, Germany) and LASAGNA-Search 2.0 [21]. In addition, intronic regions of interest were selected according to ChIP-seq data by Massie et al. [22]. The other putative AR-binding sites on CERK (−8600 bp from TSS) and CHKA (Intron 2, 27,500 bp from TSS) genes were selected according to ChIP-seq data by Wilson et al. [23] and Camacho et al. [24].

Proliferation assay: Assessment of proliferation was conducted using the IncuCyte system after MALAT1 gapmer delivery or CHKA inhibitor treatment (Hexadecyltrimethylammonium bromide (CHKAi, Merk Millipore, Burlington, MA, USA, #219374) [5] at times and doses indicated in figure legends. Cells were seeded at 35,000 cells/well in quadruplicate on a 24-well plate (Corning), with IncuCyte readings taken at six h-cycles starting from day 0 (16 images per well). The IncuCyte algorithm was used for phase area confluence ratio calculations.

Cell death assay: Apoptosis was determined using a Cell Death Detection ELISA PLUS kit (Roche, Basel, Switzerland) with cell lysates following the manufacturers’ instructions. Absorbance at 405 nm and 490 nm was measured using VICTOR X4 (Perkin Elmer).

Intracellular and extracellular samples for NMR Analysis: Cell pellets and related cell culture media were stored at −80 °C until metabolomic analysis was performed by NMR spectroscopy. Then, the shots were resuspended in ice-cold extraction solvents (methanol/chloroform/water (1:1:1)) and vigorously vortexed for intracellular metabolome. At least 24 h later, polar and lipid phases were separated by centrifugation at 20,000× *g* at 4 °C for 30 min. Next, the polar methanol/water phase containing water-soluble cellular metabolites was lyophilized using a rotary evaporator (Savant RTV 4104 freeze dryer). In contrast, the organic phase (lipid phase) was collected in a tube, evaporating chloroform under nitrogen gas flow. Both phases of extracted cells were stored at −20 °C. For extracellular metabolome, cell culture medium extraction was performed by adding ice-cold extraction solvent (ten volumes of ethanolic solution (EtOH:H_2_O, 77:23, *v*/*v*)) to each tube and stored at −20 °C for at least 24 h. Afterward, the samples were centrifuged at 14,000× *g* for 30 min and the supernatant obtained was then freeze-dried in a Savant RTV 4104 freeze dryer. The aqueous fractions from cells and extracellular media were reconstituted in 700 μL D_2_O using TSP (0.1 mM) as NMR internal standards. In contrast, lipid fractions from cells were resuspended in a CD3OD/CDCl3 solution (2:1 *v*/*v*) with 0.05% of tetramethylsilane (TMS) as an internal reference [25]. 

Metabolomics analysis by NMR spectroscopy: Deuterated reagents (methanol (CD_3_OD), chloroform (CDCl3) and deuterium oxide (D_2_O) (Cambridge Isotope Laboratories, Inc.), and 3-(trimethylsilyl) propionic-2,2,3,3-d4 acid sodium salt (TSP) (Merck & Co., Montreal, QC, Canada) were used for NMR analyses. High-resolution 1H-NMR analysis was performed at 25 °C at 400 MHz (9.4 T Bruker AVANCE spectrometer; Karlsruhe, Germany, Europe) on aqueous and organic cell extracts using acquisition pulses, water pre-saturation, data processing, and peak area deconvolution, as previously described [26]. The absolute quantification of aqueous metabolites, determined by comparing the integral of each metabolite to the integral of reference standard TSP and corrected by respective proton numbers for metabolite and TSP, was expressed as nanomoles/10^6^ cells (nmol/10^6^) [27]. The relative quantification of lipid metabolites was expressed by % determined by individual metabolite/all metabolites investigated. Due to the large number of cells needed for NMR-based metabolomics studies (>10^6^ cells/sample) for statistical analyses, we considered a pool (*n* = 3) of PCa cell lines (PC3, *n* = 2; and C27IM, *n* = 1). We reported the mean concentration (nmol/10^6^ cells) of individual metabolites for both cell lines in Table S1. Quantitative enrichment analysis identifies biologically meaningful patterns enriched in quantitative metabolomic data. MetaboAnalyst 4.0 (Free web server available at http://metaboanalyst.ca, accessed on 13 April 2022) was used to identify the biochemical pathways affected by MALAT1 depletion determined by quantitative enrichment analyses. 

RNA ImmunoPrecipitation (RIP): RIP was performed in LNCaP (*n* = 2) and HUVEC (*n*= 2) cells as described in [28]. A specific antibody to AR was used (C-19, Santa Cruz #815). The negative control was the absence of antibodies (NoAb). In addition, lncRNA recovery was analyzed as described in Aiello et al. [19].

Immunofluorescence and confocal microscopy: LNCaP cells were fixed in 4% paraformaldehyde and were maintained for 1 h at room temperature in blocking buffer (PBS, 10% Goat Serum). Cells were incubated overnight with anti-AR antibody (441; sc-7305), diluted 1:50 in PBS 5% Goat Serum, and then 45 min at room temperature with anti-mouse Alexa Fluor 546 (ThermoFisher, Waltham, MA, USA, A11003), diluted 1:200 in 5% Goat Serum in PBS, Nuclei were counterstained with DAPI. Images were taken using a Nikon Eclipse Ti2 confocal microscope and Z stack images were processed by NIS Elements AR 5.30 software (Nikon Europe B.V.) using the same acquisition settings. Representative fields were further analyzed with ImageJ software (National Institute of Health) to show red fluorescence intracellular distribution in control conditions and after DHT treatment or exposure to LACZ and MALAT1 gapmers.

Statistical analysis: Data were expressed as mean ± SEM or fold change, as indicated in figure legends. Statistical significance was calculated using a parametric paired two-tailed Student’s *t*-test and a one-way ANOVA test (Bonferroni’s multiple comparisons test). Statistical analysis was performed using GraphPad Prism 8.0 software or Excel. *p*-values ≤ 0.05 were considered significant. 

## 3. Results

### 3.1. MALAT1 Targeting Changes Metabolites of the Choline Pathway in PCa Cells

**Figure 1:**
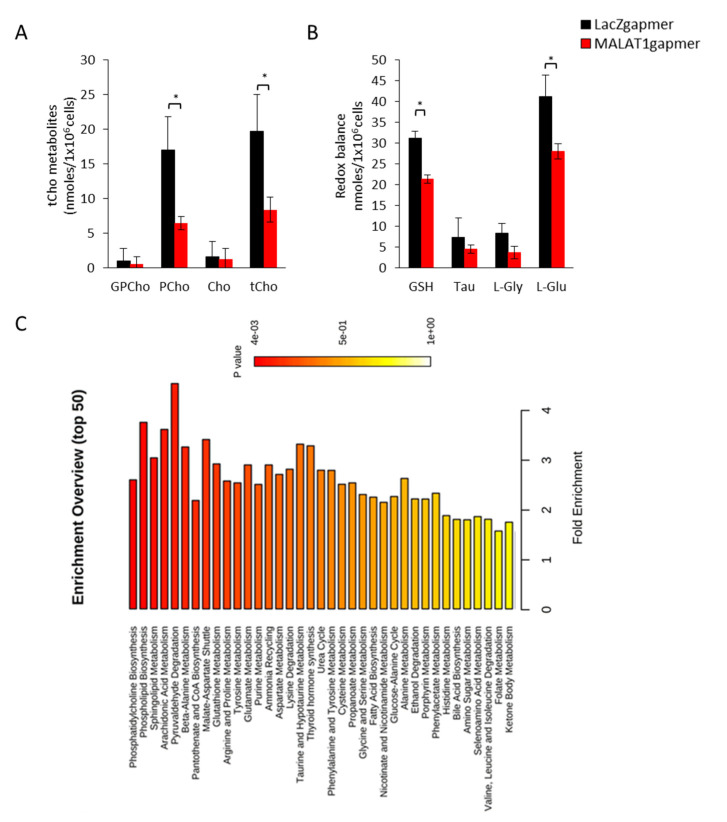
Metabolomic analysis in MALAT1-depleted PCa cell lines: (**A**,**B**) Quantitative H-1NMR spectroscopy of Phosphatidylcholine metabolites in MALAT1-depleted and LacZ control PCa cells. Phosphatidylcholine metabolites (choline, Cho; phosphocholine, PCho; glycerophosphocholine, GPC, (**A**) and Redox Balance metabolism (GSH, Glutathione; Tau, Taurine; L-Gly, L-Glycine; L-Glu, L-Glutamic acid, (**B**) are expressed as mean ± SEM (N = 3). * *p* < 0.05 vs. gapmer LacZ. (**C**) Quantitative Enrichment Analysis of the biologically meaningful patterns significantly altered the whole metabolome obtained by quantitative NMR-based spectroscopy in MALAT1-depleted vs. LacZ control cells.

The impact of MALAT1 targeting on the metabolic function of PCa cells was analyzed by quantitative High-resolution 1H Nuclear Magnetic Resonance (NMR) spectroscopy in MALAT1-depleted vs. LACZ PCa control cells after 48 h from gapmer delivery. In the presence of MALAT1 gapmers, we found a significant decrease in the intracellular levels of total choline-containing metabolites (tCho) (Figure 1A). The resonances originating from tCho include signals of several phosphatidylcholine metabolites. They include choline (Cho), phosphocholine (PCho), and glycerophosphocholine (GPC), revealing a significant reduction of the PCho metabolite (*p* = 0.02). In addition, we found a significant decrease in glutathione (*p* = 0.03) and its precursor L-glutamic acid (*p* = 0.04), a decrease in the average content of glycine, which is involved in the redox balance, and a reduction in the antioxidant taurine (*p* = 0.07 and *p* = 0.06, respectively, Figure 1B). Conversely, no significant changes in other metabolites involved in energy production or amino acid and nucleotide metabolism were observed (see Table S1). Quantitative enrichment analysis was performed on the metabolites obtained by NMR-based spectroscopy in MALAT1-depleted vs. LacZ PC3 and C27IM cells (Figure 1C). Phosphatidylcholine, Phospholipid biosynthesis, Sphingolipids, and arachidonic acid metabolism were the most enriched pathways associated with MALAT1 targeting (Figure 1C). This finding is in agreement with the reduced content in PCho and glutathione.

### 3.2. MALAT1 Targeting Decreases CHKA Gene Expression in PCa Cells

**Figure 2:**
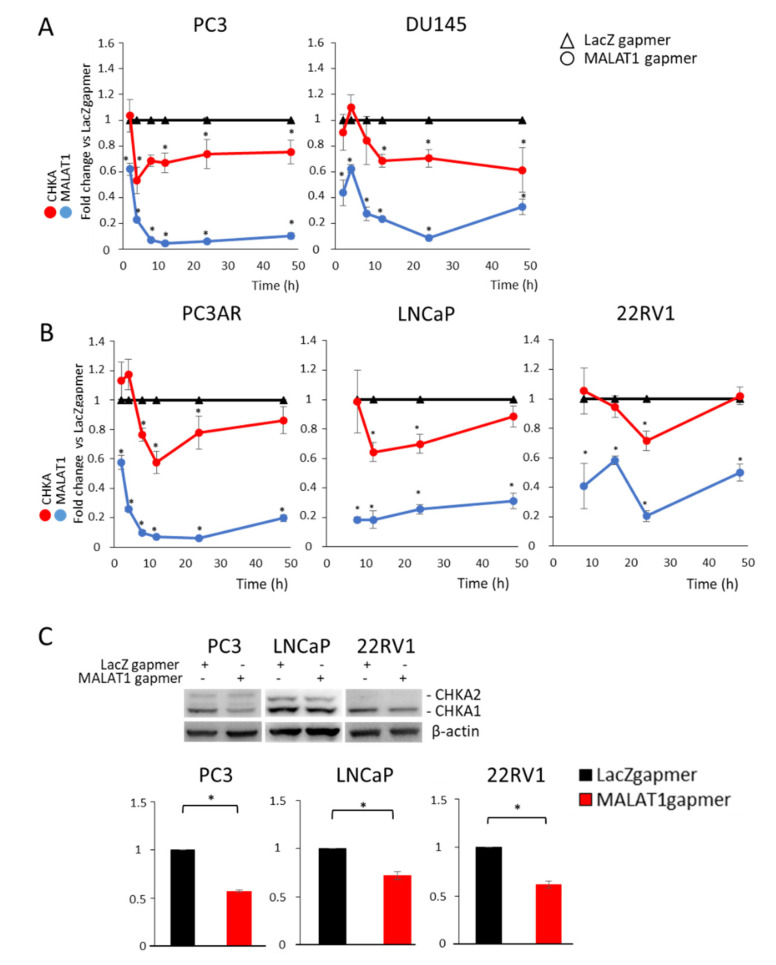
Effects on Choline kinase alpha (CHKA) expression in MALAT1-depleted PCa cells: (**A**,**B**) Quantification of CHKA and MALAT1 transcripts by qRT-PCR in androgen-independent (PC3 and DU145, (**A**), androgen-sensitive (PC3AR and LNCaP, (**B**), and androgen refractory (22RV1, (**B**)) cell lines after transfection with specific (MALAT1) or control (LacZ) gapmers at different time points. LNCaP and 22RV1 cells were cultured with hormone-deprived (SS) serum. (**C**) Representative CHKA western blot and densitometry analysis in PC3, LNCaP, and 22RV1 cells after MALAT1 depletion (24 h in PC3 and 22RV1 and 16 h in LNCaP cells). β-actin was used as a loading control. Data represented as mean of fold change vs. LacZgapmer ± SEM (N = 3). * *p* < 0.05 MALAT1gapmer vs. LacZgapmer.

Consistent with the metabolites screening, CHKA, which catalyzes the conversion of choline to phosphocholine along the Kennedy pathway, emerged among the modulated genes identified in a MALAT1-dependent transcriptome analysis performed in advanced and metastatic PCa cell lines [18]. In order to investigate the consequences of MALAT1 targeting on the expression of CHKA, we first analyzed CHKA mRNA by qPCR in five PCa cells chosen according to the differences in their level of AR expression. Specifically, PC3 and DU145 are deleted for AR (AR-null cell lines), PC3AR, and LNCaP express full-length AR (AR-FL), and 22RV1 co-expresses the AR-FL and AR variant ARV7, which lacks the AR Ligand Binding Domain. Figure 2A,B shows that the downregulation of CHKA appeared early upon MALAT1 silencing and was prolonged up to 48 h in the AR-null cell lines compared to AR-positive cells (Figure 2A,B; the blue line represents the MALAT1 transcript). In AR-positive cells (PC3AR, LNCaP, and 22RV1), the CHKA expression profile (red line) exhibited repression compared to control (LacZ gapmers) in a time window ranging between 12 and 24 h either in cells cultured in standard (data not shown) or hormone-deprived medium (Figure 2B). Similarly, CHKA protein levels decreased in PC3, LNCaP, and 22RV1 cells (Figure 2C). 

**Figure 3:**
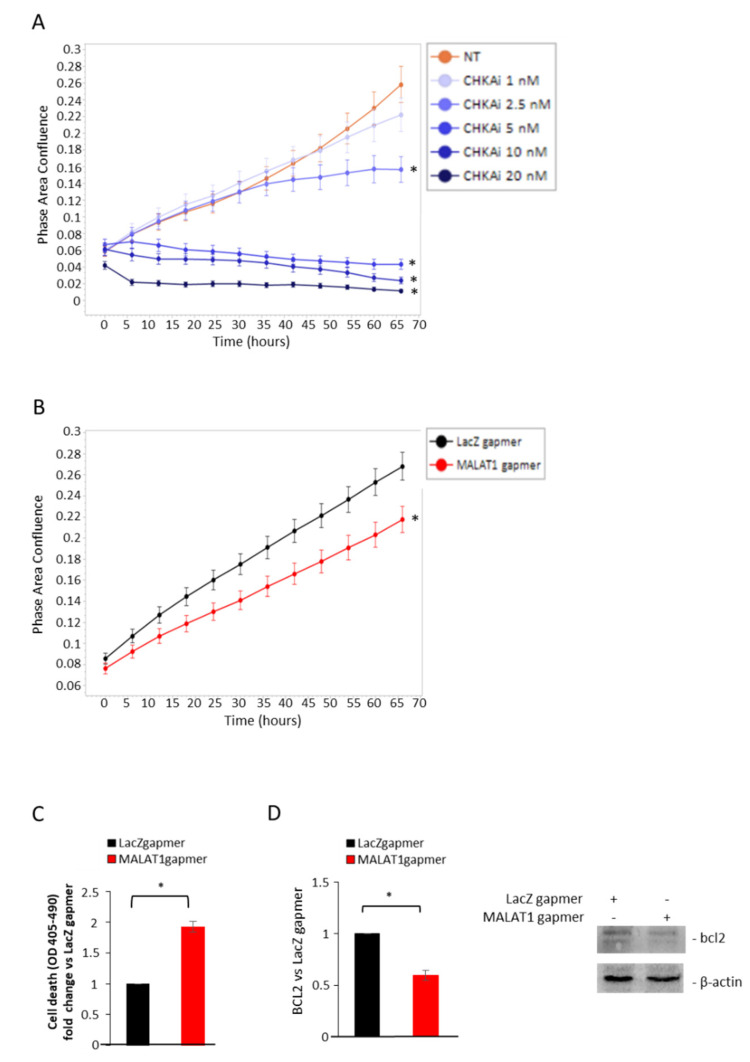
Effects on cell proliferation and cell death upon MALAT1 depletion or CHKA inhibitor treatment in LNCaP cells: (**A**) LNCaP cells were treated with CHKAi at the dose and time indicated and monitored using the IncuCyte live cell analysis system. Cell confluence was calculated from raw data images; data represent mean ± SEM of *n* = 2 independent experiments, each performed in quadruplicate. * *p* < 0.05 vs. NT. (**B**) Cell proliferation after MALAT1 gapmer delivery was monitored using the IncuCyte live cell analysis system. Cell confluence was calculated from raw data images; data represent mean ± SEM of *n* = 2 independent experiments, each performed in triplicate. * *p* < 0.05 vs. LacZgapmer. (**C**) Apoptosis induction by MALAT1 depletion (96 h) evaluated using Cell Death Detection ELISA Kit (Roche, Palo Alto, CA, USA) as described in Methods. Data are expressed as fold change vs. LacZ gapmer, representing the mean ± SEM of three independent experiments. * *p* < 0.05 MALAT1gapmer vs. LacZgapmer. (**D**) Representative blc2 western blot and densitometry analysis after MALAT1 depletion (72 h). β-actin was used as a loading control. Data are represented as mean of fold change vs. LacZgapmer ± SEM (*n* = 3). * *p* < 0.05 MALAT1 gapmer vs. LacZ gapmer.

Because CHKA is involved in the phosphatidylcholine biosynthesis, we evaluated whether other genes belonging to the phospholipid and sphingomyelin metabolism were modulated under the same experimental conditions. Specifically, the analysis included phospholipase C gamma 1 (PLCG1), sphingomyelin phosphodiesterase 1 (SMPD1, and Ceramide kinase (CERK). As shown in Figure S1, MALAT1 targeting did not substantially alter PLGC1, SMPD 1, or CERK gene expression in the AR-null cell lines (PC3 and DU145). Consistently, CHKA pharmacological inhibition (Figure 3C) led to a dose-dependent reduction in LNCaP cell growth over time, about 40% at 2.5 nM and 90% at higher concentrations, specifically, 5, 10, and 20 nM, peaking after 65 h of treatment (Figure 3A,B). On the other hand, MALAT1 targeting caused a 25% reduction in proliferation rate, a two-fold increase in apoptosis as measured by cell death assay, and a decrease in Bcl2 expression (Figure S2). These results indicate that perturbation of the MALAT1 or CHKA axis reduces the oncogenic phenotype of LNCaP cells, leading to cell growth inhibition and apoptosis. 

### 3.3. MALAT1 Targeting Determines Chromatin Remodeling on CHKA Genomic Region

**Figure 4:**
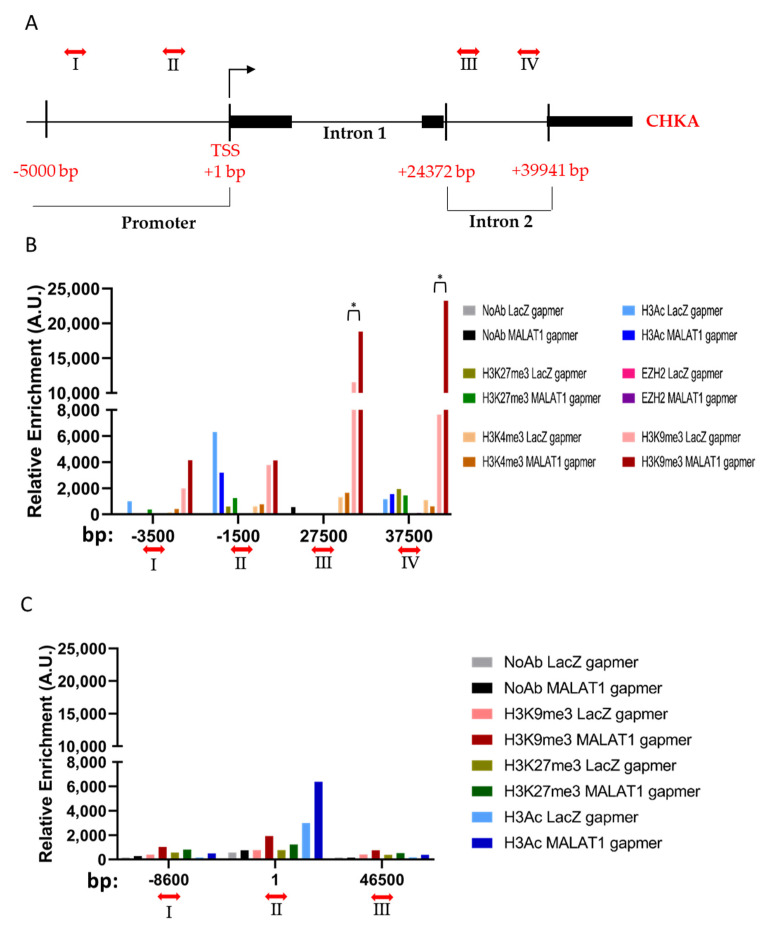
Chromatin remodeling in CHKA and Ceramide kinase (CERK) genes upon MALAT1 targeting: (**A**) Schematic diagram of CHKA genomic regions. Double-arrowed lines and roman numbers identify primer position among CHKA genomic regions; arabic numerals refer to the genomic positions in base pairs; black boxes refer to exons of the CHKA gene. (**B**) EZH2 chromatin binding and H3Ac, H3K27me3, H3K4me3, and H3K9me3 histone tail modifications on CHKA genome regions after MALAT1 gapmers transfection (16 h). (**C**) H3Ac, H3K27me3, and H3K9me3 histone tail modifications on CERK genome regions after MALAT1 gapmer transfection (16 h). No Antibody (NoAb) served as a negative control. Data are represented as relative enrichment in Arbitrary Units (AU). * *p* < 0.05 MALAT1gapmer vs. LacZgapmer.

In order to dynamically map the most common epigenetic modifications along the specific genomic regulatory regions of CHKA (see schematic diagram in Figure 4A), we performed a ChIP assay in PC3 cells before and after MALAT1 targeting. Potential EZH2 recruitment or enrichment in histone modifications, including histone H3 pan-acetylation (H3Ac), histone H3 trimethylation at lysine (K) residues 27, 4, and 9 (H3K27me3, H3K4me3, and H3K9me3) was investigated at the 16 h time-point upon MALAT1 or LacZ gapmers delivery (Figure 4B). The no-antibody (NoAb) condition served as a negative control. MALAT1 targeting determined a significant enrichment of the repressive H3K9me3 modification within the CHKA intron two sequence (primers designed as III and IV), a genomic region known for AR putative binding sites [5,22]. A trend in terms of decrease in pan-acetylated histone H3 was appreciated at −1500 base pairs (bp) from the transcription starting site (TSS) of the CHKA gene promoter, which is consistent with the repression of CHKA mRNA observed at that time window (Figure 2A,C). No changes were observed in the level of H3K27me3 or its methylase EZH2 and H3K4me3 enrichment.

Another kinase relevant to the sphingolipid homeostasis, namely, the ceramide kinase (CERK), has been recently identified as an AR target [24]. Therefore, CERK genomic regions were explored by ChIP as an internal control reference (Figure 4C). No significant changes in histone modification levels were found in this chromatin context, in line with the unaltered expression of CERK mRNA in PC3 cells upon MALAT1 targeting (Figure S1B).

Overall, these data indicate that in MALAT1-depleted cells the chromatin landscape enriches repressive signals, contributing to CHKA mRNA reduction.

### 3.4. Effects of MALAT1 Targeting Combined with Dihydrotestosterone (DHT) Treatment on Metabolic Genes Involved in Phospholipid/Sphingolipid Homeostasis

Previous studies [5,22] have shown that CHKA is a clinically relevant AR gene target. In these reports, ChIP-Seq detected strong intragenic AR binding sites. Herein, we asked whether MALAT1 targeting could have a different impact in PCa cells cultured in the presence or absence of androgen. In order to address this question, we used LNCaP, which endogenously expresses AR full length (AR-FL) and 22RV1 cells, in which the AR variant ARV7 (lacking the AR Ligand Binding Domain) co-exists with AR-FL. LNCaP or 22RV1 were transfected with gapmers targeting MALAT1. 

**Figure 5:**
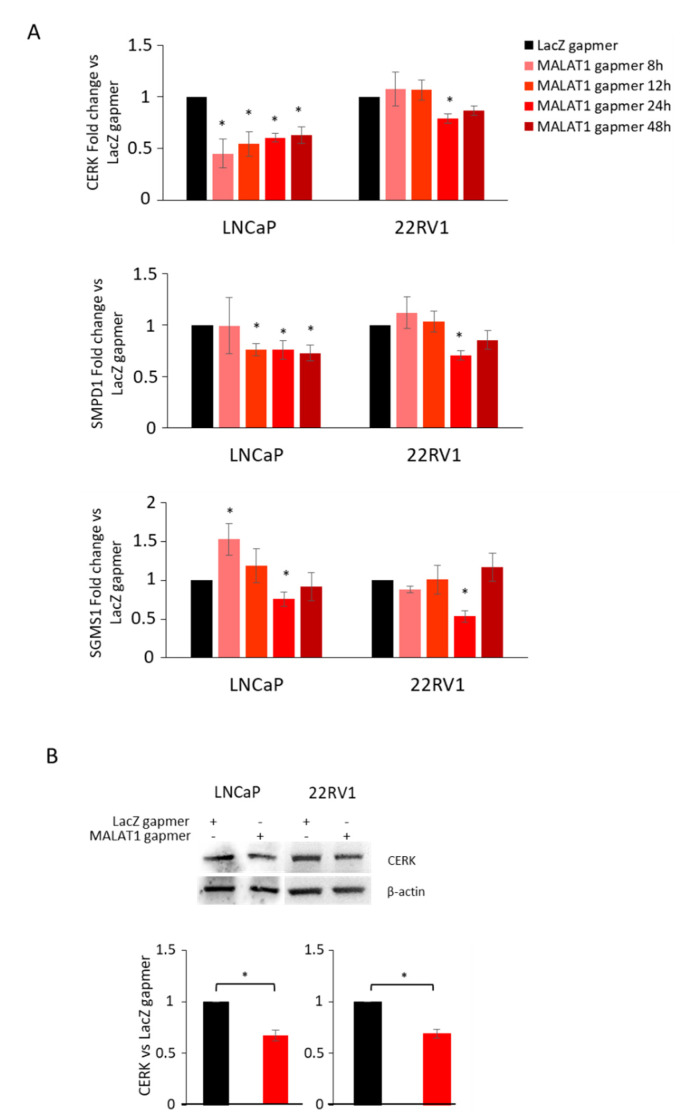
Effects on sphingolipids/ceramide enzymes in MALAT1-depleted PCa cells cultured with hormone-deprived (SS) serum: (**A**) CERK, SMPD1, and SGMS1 transcripts quantified by qRT-PCR in LNCaP and 22RV1 cells grown were grown in SS condition after transfection with specific (MALAT1) or control (LacZ) gapmers at different time points. (**B**) Representative CERK western blot and relative densitometry analysis in LNCaP and 22RV1 cells upon 16 h and 24 h, respectively, from transfection with specific (MALAT1) or control (LacZ) gapmers. Β-actin was used as a loading control. Data are represented as the mean of fold change vs. LacZgapmer ± SEM (N = 4). * *p* < 0.05 MALAT1gapmer vs. LacZgapmer.

As CERK kinase is under the control of AR, we measured the expression of CERK before and after MALAT1 targeting in cells cultured in a hormone-deprived medium. CERK mRNA was efficiently and stably silenced upon MALAT1 targeting in LNCAP. In 22RV1, however, this effect was evident at 24 h upon MALAT1 gapmer delivery (Figure 5A). A similar impact upon MALAT1 targeting was observed for SMPD1 and SGMS1 (Figure 5A). A significant decrease in CERK protein level was found in both LNCaP and 22RV1 cells (Figure 5B).

To realize MALAT1 targeting plus DHT combination treatment, a DHT dose-dependent experiment ranging from 10^−8^ M to 10^−6^ M was carried out in both cell lines. The expression of CHKA and CERK, the latter having been recently identified as an androgen-repressed gene, and of PSA, a classical AR-positive regulated gene, was analyzed by qRT-PCR. In LNCaP cells, CHKA mRNA increased more than three-fold, although only at the highest dose of DHT, whereas PSA and CERK were significantly modulated at a physiological concentration (10^−8^ M). In 22RV1 cells, a defective androgen sensitivity was observed, which can reasonably be attributed to the presence of the ARv7 variant. Consequently, we observed a small increase in CHKA and PSA regardless of DHT concentration in this model, while CERK was modulated at the lowest dosage (Figure S3A,B).

**Figure 6:**
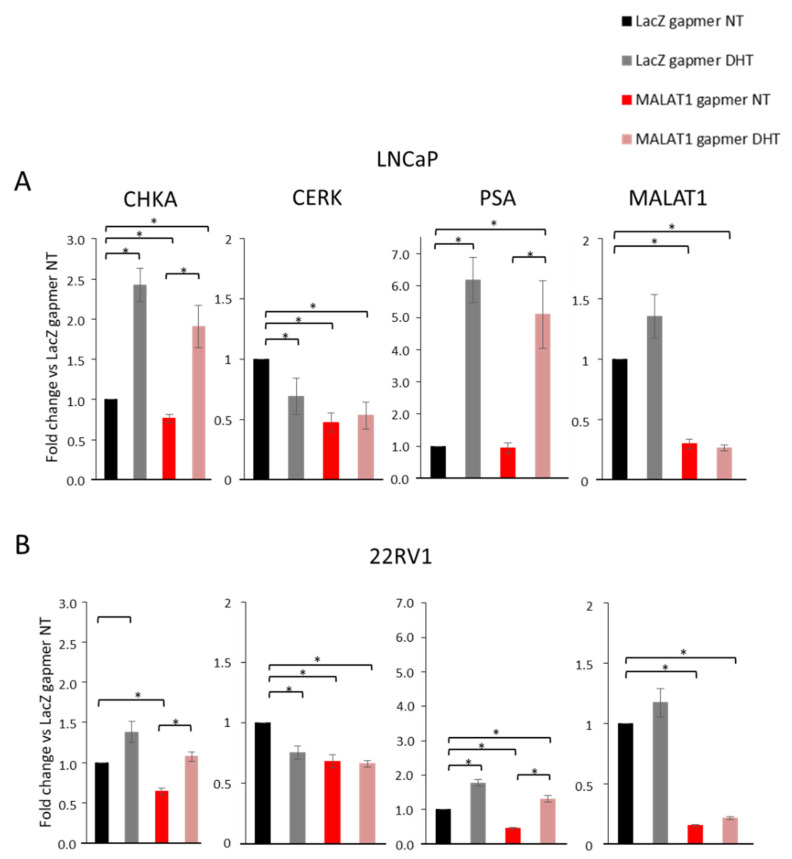
Effects on CHKA expression in MALAT1-depleted PCa cells combined with Dihydrotestosterone (DHT) treatment: (**A**,**B**) CHKA, CERK, Prostate-specific antigen (PSA), and MALAT1 transcript levels quantified after MALAT1 gapmer transfection in LNCaP (16 h of transfection), (**A**) and 22RV1 (24 h of transfection), (**B**) cells treated with or without DHT (10^–6^ M for LNCaP and 10^–8^ M for 22RV1) for 16 h. Data are represented as mean of fold change vs. LacZgapmer ± SEM (LNCaP N = 5; 22RV1 N = 4). * *p* < 0.05.

In the combined treatment (Figure 6), LNCaP and 22RV1 cells transfected with MALAT1 or LacZ gapmers were treated with DHT (10^−6^ M for LNCaP and 10^−8^ M for 22RV1) for 16 h, focusing on CHKA gene modulation. As a control, we examined the effect of MALAT1 targeting and androgen treatment on the CERK gene as an androgen-repressed target, and in parallel on PSA mRNA as an androgen-induced target. In this setting, LNCaP cells transfected with LacZgapmers preserved androgen responsiveness in terms of CHKA and PSA mRNA increase (2.5- and six-fold induction by LacZgapmers DHT vs. LacZgapmers NT) and CERK mRNA downmodulation (40% repression LacZgapmers DHT vs. LacZgapmers NT), which is in line with the data reported by Camacho et al. [24]. 

MALAT1 targeting reduced basal level of CHKA and CERK mRNA, as shown in Figure 2 and Figure 5, whereas no modulation of PSA was found in LNCaP cells. In addition, MALAT1 targeting did not alter the induction of CHKA or PSA mRNA mediated by androgen treatment, preserving a consistent increase vs. MALAT1 gapmers alone. Similarly, MALAT1 targeting and DHT treatment did not enhance CERK mRNA inhibition in the same cells. 

In 22RV1 cells (expressing the AR-V7 variant), we noted an overall reduced androgen responsiveness compared to LNCaP cells in all experimental conditions (Figure 6A vs. Figure 6B). In 22RV1 cells, PSA appeared to be repressed upon MALAT1 targeting at the 24 h time point.

Upon MALAT1 targeting, CHKA and CERK protein levels paralleled those observed in mRNA in terms of inhibition in both LNCaP and 22RV1 cells (Figure S4). However, this time point corresponds to maximum efficiency in mRNA silencing after MALAT1 targeting (see Figure 2). Here, a stabilization of CHKA protein by DHT was only partially observed despite induction of AR protein levels (two-fold vs. LacZ gapmers in LNCaP and 22RV1 cells) and reduction of CERK protein level (30% in LNCaP). 

Altogether, these results suggest that the androgen responsiveness characterized by a complete response with AR full length or by a weak response with the ARv7 variant was preserved regardless of MALAT1. However, the consequence of MALAT1 depletion was particularly evident on the androgen-repressed target gene, CERK, and on the high-dose androgen positive-regulated gene, CHKA.

### 3.5. Dynamic Recruitment of AR on CHKA Genomic Sequences before/after MALAT1 Targeting in the Presence or Absence of DHT

**Figure 7:**
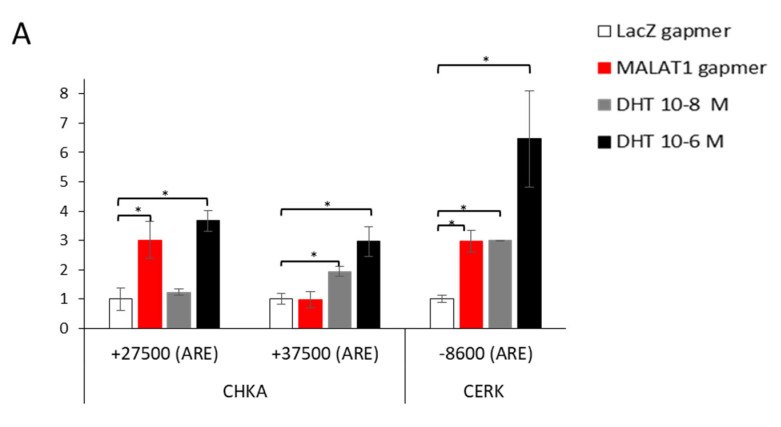

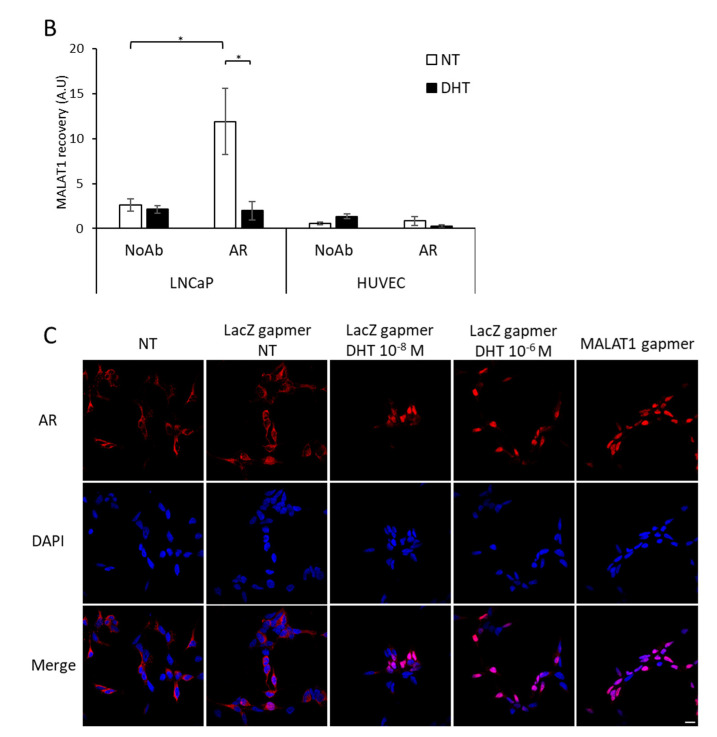
AR recruitment by ChIPs on CHKA and CERK regulatory regions and AR nuclear translocation upon MALAT1 targeting or dihydrotestosterone (DHT) treatment and MALAT1/AR interaction by RIP: (**A**) AR recruitment onto CHKA ARE located at +27,500 and +37,500 bp from TSS (Intronic region) and CERK ARE located at −8500 bp from TSS or after MALAT1 gapmer transfection (16 h) or DHT treatment (10^–8^ M and 10^–6^ M 4 h). No Antibody (NoAb) served as a negative control. Data represented as the mean of AR fold change after subtraction of NoAb ± SEM (N = 2). * *p* < 0.05 vs. gapmer LacZ. (**B**) MALAT1 interaction with AR in LNCaP and HUVEC cells (used as control) before and after DHT (10^–7^ M, 1 h) treatment detected by RIP assay. RIPs were performed using antibodies specific to AR or in the absence of Ab (NoAb) as a negative control. RNA was recovered and analyzed by qRT-PCR. Data are represented as mean ± SEM of two independent experiments performed in duplicate. * *p* < 0.05. (**C**) Androgen receptor subcellular localization was evaluated by confocal microscopy upon MALAT1 gapmer delivery (16 h) or DHT treatment (10^−8^ M or 10^−6^ M for 4 h). Scale bar: 15 μm.

In order to investigate this aspect further, we performed ChIP experiments for AR recruitment on CHKA and CERK regulatory regions at the 16 h time point upon MALAT1 or LacZ gapmer delivery (Figure 7A). The no-antibody (NoAb) condition served as a negative control. Of interest, upon MALAT1 targeting AR was tightly recruited to CHKA regulatory region, specifically, at the ARE in intron-2 described by Wilson et al. [23] and not in the ARE located at +35,000 bp from TSS (Asim et al. [5]), designated as III and IV, respectively, in Figure 4A,B. In parallel, AR recruitment was observed at the ARE site on the CERK promoter described by Camacho et al. [24] (Figure 7A, designed as I in the schematic of the CERK regulatory genomic region in Figure 4C). AR recruitment after MALAT1 depletion paralleled that obtained with the androgen treatment. DHT stimulation at both concentrations, 10^−8^ M and 10^−6^ M, increased the AR recruitment at the same genomic regulatory regions in both genes. This suggests that in cells depleted of MALAT1, AR recruitment is facilitated.

These data prompted us to evaluate whether MALAT1 might associate with AR to act as a co-regulator and eventually affect the transcription of AR-dependent genes. To address this possibility, we immunoprecipitated the AR in LNCaP cells treated with androgens and analyzed whether MALAT1 was co-immunoprecipitated by RNA immunoprecipitation (RIP). AR appeared to associate with MALAT1 in basal conditions (Figure 7B). However, upon DHT treatment, this interaction was abrogated. No such association was elicited in the HUVEC cells used as a control reference cellular context, regardless of DHT stimulation. 

We queried whether the AR might translocate into the nucleus upon MALAT1 targeting in order to deepen insight into the underlying mechanism. We analyzed the AR shuttling from cytoplasm to nucleus in LNCaP cells before/after MALAT1 targeting using confocal microscopy (Figure 7C). As negative or positive reference controls, we included proliferating (NT) and DHT-treated LNCaP cells (10^–8^ M and 10^–6^ M, respectively). Figure 7C depicts cytoplasm–nuclear translocation of AR upon MALAT1 targeting comparable to that achieved upon DHT at both concentrations. Accordingly, nuclear cytoplasmic fractionation, observed with western blotting, increased AR in the nuclear compartment (1.9-fold vs. NT), which was mirrored by a decrease in the cytoplasm upon MALAT1 gapmer delivery of LacZ gapmers (0.7-fold vs. NT, Figure S5). In addition, MALAT1 targeting increased the H3k27me3 level at both CHKA and CERK genomic regulatory regions, thus substantiating the repressive chromatin conformation (Figure S5B). These data suggest that MALAT1 is an active regulator of AR-mediated signaling.

## 4. Discussion 

### 4.1. CHKA Is a Target of MALAT1

The present work significantly establishes CHKA, an essential gene in phospholipid metabolism, as a MALAT1 target. In our previous work [18], the transcriptomic analysis performed in advanced or metastatic PCa cell lines and OSCs upon MALAT1 targeting revealed CHKA among a subset of differentially-expressed genes associated with metabolic reprogramming. Accordingly, the NMR spectroscopy significantly altered the choline/PC metabolism, placing MALAT1 targeting as a potential therapeutic option for PCa. Interestingly, CHKA expression is androgen-regulated in cell lines, xenografts, and human tissue and is positively associated with Asim’s tumor stage [5]. Indeed, different studies have shown the role of CHKA in human PCa; however, the molecular mechanisms have not yet been fully elucidated. Recently, in the human DU145 PCa cell line, the presence of a molecular complex involving FGFR1 and CHKA was described by [29]. FGFR1 promotes PCa progression by dysregulating choline metabolism and CHKA [29]. In this light, the crosstalk between FGFR1–choline metabolism might represent an additional potential target for managing PCa progression.

Moreover, CHKA is involved in antioxidant cellular defenses by regulating glutathione, cysteine content, and reactive oxygen species level. In ovarian cancer, the antioxidant role of CHKA, which is exerted by decreasing glutathione cysteine and methionine content, contributes to sensitizing cancer cells to chemotherapy [30,31]. In our hands, MALAT1 was found to modulate CHKA expression, PCho, and glutathione in PCa cells, revealing a new MALAT1-dependent link between PC biosynthesis and redox balance in cancer cells. 

In hepatocellular carcinoma [32], MALAT1 knockdown inhibited glucose uptake and lipogenesis by reducing the expression levels of lipidic metabolism-related genes. This condition contributes to the oncogenic role of MALAT1 in tumor cell proliferation and invasion. In this light, following MALAT1 depletion we observed a metabolic reprogramming involving PC, redox balance, and sphingolipid metabolism. In fact, in addition to the well-known role of CHK in PC metabolism, the CHKA substrate phosphocholine influences bioactive sphingolipid level and activity, playing a critical role in different biological processes such as growth regulation and cell migration, adhesion, apoptosis, senescence, and inflammatory response [33]. Pharmacological or genetic depletion of CHKA induces cell growth arrest in both in vivo and in vitro experimental models by altering key players in oncogenic cell signaling. On the other hand, the inhibition of cell signaling (e.g., PI3K) inhibits expression of CHKA, highlighting the role of choline kinase in cancer cell transduction and oncogenic metabolic reprogramming [10,16,34,35,36] (and see references therein). This supports the role of bioactive sphingolipids in cancer progression [33]. Lastly, Sphingomyelin (SM), the most abundant sphingolipid in mammalian cells, can be degraded into Ceramide and Phosphocholine by sphingomyelinases. 

Conversely, sphingomyelin synthases (SGMSs) use Phosphatidylcholine and Ceramide to form SM and diacylglycerol (DAG) [37]. In particular, Sphingomyelin and Ceramide play opposite roles in cell death, survival, and proliferation. Specifically, high levels of Sphingomyelin lead to survival, migration, proliferation, and inflammation. Instead, high levels of Ceramide induce cell death and cell cycle arrest [38]. Of note, SGMS and CERK are involved in sphingolipid homeostasis. CERK and its product, ceramide 1-phosphate (C1P), regulate cell growth, death, and cell migration/invasion in different cancers, including PCa [24]. In cancer cells, low levels of Ceramide are maintained by high SGMS activity, which reduces ceramide-induced cell death [38].

On the other hand, the CERK-dependent phosphorylation of Ceramide enhances its role in proliferation and migration. Furthermore, CERK activity and its metabolites support cancer progression, particularly PCa cell aggressiveness [24]. In this scenario, our data indicate that both kinases, being sensitive to AR signaling, are modulated by MALAT1 targeting. The cholinic phenotype abrogation is clinically relevant (see cartoons in the graphical abstract).

### 4.2. Lipid Metabolism Is under the Control of AR Signaling

**Figure 8:**
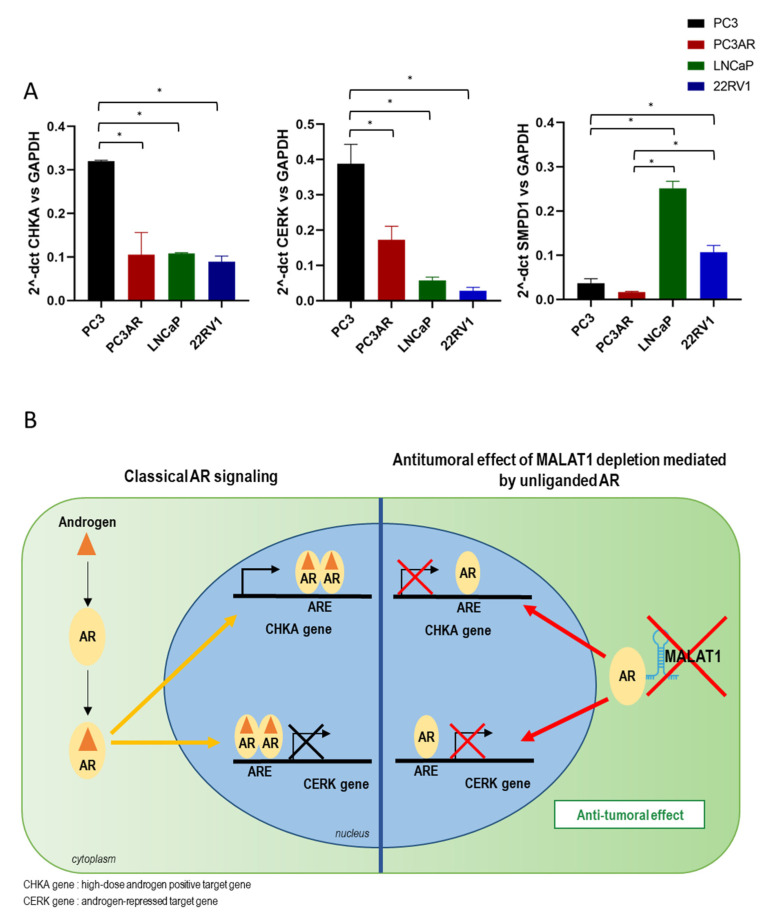
The basal level of CHKA, CERK, and SMPD1 mRNA in different PCa cell lines: (**A**) Quantification of CHKA, CERK, and SMPD1 by qPCR. Data were normalized to GAPDH expression. Data are represented as the mean of 2-ΔCt ± SEM (*n* = 3). * *p* < 0.05. (**B**) The antitumoral effect of MALAT1 targeting. Schematic cartoon showing the role of MALAT1 in androgen receptor signaling in advanced prostate cancer. The metabolic genes CHKA and CERK are transcriptionally regulated by androgen/androgen receptor (AR) on Androgen Responsive Element (ARE) sites (Left). MALAT1 reduces AR activity by forming a MALAT1/AR complex without androgens. MALAT1 depletion enables unliganded-AR to regulate CHKA and CERK genes, exerting anti-tumoral activity (Right).

Lipogenesis and lipid catabolism is controlled by AR signaling. Lipid biosynthesis promotes CRPC development, counteracting the effect of AR antagonist treatment [24,39]. Furthermore, the lipid-related enzyme level, including CHKA, CERK, and SMPD1, seems to be associated with AR expression (Figure 8A). Indeed, CHKA and CERK expression levels are higher in AR null cells than in PC3AR, LNCaP, and 22RV1. SMPD1 shows a mirrored trend, possibly associated with its role in sphingomyelin catabolism opposite SGMS, which acts as a pro-tumoral gene [38]. Therefore, as lipogenesis and lipid metabolism play a pivotal role in PCa progression by fueling membrane material and steroid hormone precursors, MALAT1 targeting might represent a potential RNA-based therapy in combination with third-generation AR antagonists by affecting CHKA/SGMS (Kennedy pathway) and CERK/Ceramide metabolism. In keeping with this, the complex sphingomyelin network decreases SGMS1 in LNCaP upon MALAT1 targeting (Figure 5A). The metabolic step of sphingomyelin synthesis involves the phosphocholine head group transferring from phosphatidylcholine to Ceramide, and is catalyzed by the SGMS. Therefore, PCho pool reduction following CHKA depletion might negatively affect SGMS expression, and thereby the maintenance of PhosphatidylCholine/*S*phingoMyelin (PC/SM) homeostasis.

Sphingolipid metabolism is under the control of AR [24] through its regulation of CERK transcription and sphingolipid homeostasis. Therefore, PC and SM status conservation is a critical event for PCa cell biology, as their oscillations affect membrane structural homeostasis and cell proliferation/death signaling. In particular, MALAT1 might be essential in the crosstalk between the SM cycle [38] and PC cycle [40] as well as between their related mediators, Ceramide and DAG. 

### 4.3. Crosstalk between MALAT1/AR and AR Recruitment along CHKA Genomic Regions before/after MALAT1 Depletion +/− DHT

This work demonstrates that AR signaling might interact with MALAT1 in PCa cells. This evidence adds a layer of complexity to the role of MALAT1 in cancer, particularly in hormone-driven cancers such as PCa. In our hands, AR/MALAT1 interaction occurs predominantly without androgens (Figure 7B). Interestingly, we observed higher CHKA and CERK mRNA expression in AR-null than in AR-expressing PCa cell lines (Figure 7A). These results suggest AR-dependent transcriptional repression of selected target genes, e.g., CHKA and CERK, (Figure 7A and Figure 8B), detectable in the absence of ligand in cells cultured in a hormone-deprived environment. Specifically, MALAT1 targeting unveils a novel unliganded AR-dependent transcriptional repression of CHKA and CERK which exerts antitumoral activity.

Interestingly, AR is released from MALAT1 either by adding the ligand or after MALAT1 targeting (Figure 7B and Figure 8B). In this scenario, MALAT1 acts as an AR activity inhibitor, forming an AR/MALAT1 complex. The AR nuclear enrichment observed upon MALAT1 knockdown by confocal microscopy as well as in wbweb analysis reinforces and substantiates this concept. These data suggest the essential role of lncRNAs in PCa at the border between metabolism and AR signaling, *per se*, as well as upon response to androgens. In this light, therapeutic intervention to reduce MALAT1 intracellular content might restore a physiological metabolism in highly aggressive transformed cells, with resulting detrimental consequences on tumor growth.

## 5. Conclusions

In conclusion, this work reports an unprecedented role of MALAT1 in Choline metabolism with implications in Sfingolipid and Ceramide biosynthesis, as suggested by the downregulation of CHKA and CERK upon MALAT1 targeting (see Figure 7B and graphical abstract). Although the role of MALAT1 on PCa metabolism has previously been reported as impacting on the TCA cycle [18], the results reported here suggest that MALAT1 is more profoundly interconnected with the cellular metabolism, including a negative regulatory effect on the Kennedy pathway. Hence, as Choline synthesis is a crucial step toward membrane biogenesis and sphingolipid production, the MALAT1 targeting effect appears to overlap with that of CHKA inhibitors. This evidence suggests the option of a combined or temporally consequential powerful therapeutic strategy, at least in more resistant/aggressive cases of PCa. This possibility is further substantiated by the negative effect of MALAT1 targeting on CERK, implying a reduction in the synthesis of the sphingolipids. It is noteworthy that sphingolipids are essential components of the lipid raft domains of the plasma membrane, and this structural function is critical for apoptosis or cell proliferation.

Moreover, dysregulation of sphingolipids, including Ceramide, Sphingomyelin, or Sphingosine 1-phosphate, has been linked to drug resistance in different types of cancer. Hence, MALAT1 targeting might have essential antitumor consequences affecting these critical biosynthetic pathways. In conclusion, this manuscript describes a novel aspect of MALAT1 in cellular metabolism, possibly leading to innovative therapeutic strategies.

## Data Availability

The data presented in this study are available in this article and Supplementary Material.

## Supplementary Materials

The following are available online at https://www.mdpi.com/article/10.3390/cancers14122902/s1, Figure S1: Effects on Phospholipase C Gamma 1 (PLCG1), Sphingomyelin phosphodiesterase 1 (SMPD1), and Ceramide kinase (CERK) expressions in MALAT1 depleted PCa cells AR null; Figure S2: Effects on CHKA inhibitor treatment of cell proliferation in LNCaP cells; Figure S3: Effects on CHKA expression in Dihydrotestosterone (DHT) treated PCa cells; Figure S4: Effects on CHKA expression in MALAT1 depleted PCa cells in combination with Dihydrotestosterone (DHT) treatment; Figure S5: AR nuclear translocation upon MALAT1 targeting or dihydrotestosterone (DHT) treatment; Table S1: Absolute quantification (nmole/10^6^ cells) of aqueous metabolites in PCa. File S1: original Western Blot figures.
