# Supplementary material for: MALAT1 as a Regulator of the Androgen-Dependent Choline Kinase A Gene in the Metabolic Rewiring of Prostate Cancer
Source: Cancers (Basel). 2022 Jun 12;14(12):2902. doi: 10.3390/cancers14122902 (PMC9221206; doi:10.3390/cancers14122902)
Supplement: Supplementary file 1 [file cancers-14-02902-s001.zip › cancers-1705374-supplementary.pdf]

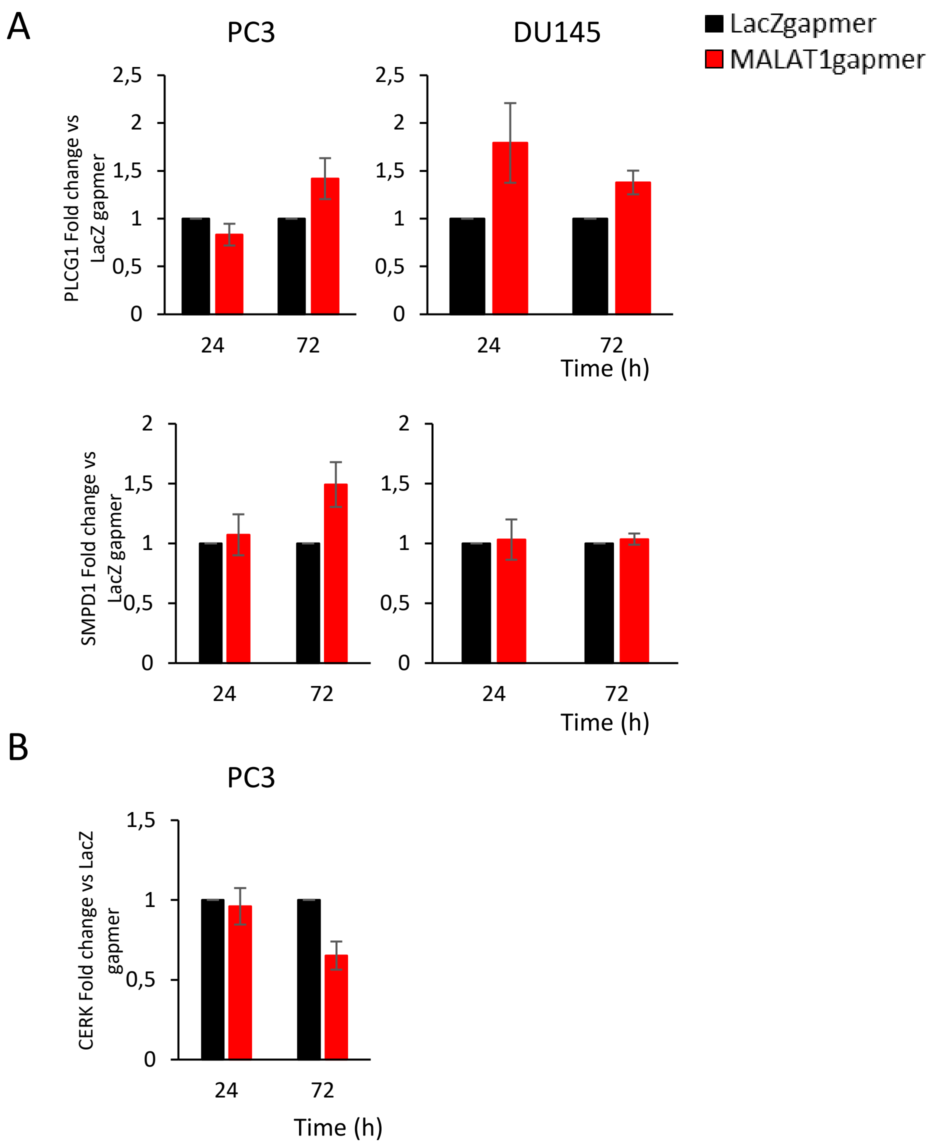

Figure S1. Effects on Phospholipase C Gamma 1 (PLCG1), Sphingomyelin phosphodiesterase 1 (SMPD1) and Ceramide kinase (CERK) expressions in MALAT1 depleted PCa cells AR null. A) PLGC1 and SMPD1 transcripts quantified by qRT-PCR in PC3 and DU145 cells after transfection with specific (MALAT1) or control (LacZ) gapmers at 24h and 72h upon gapmers delivery. B) CERK transcript quantified by qRT-PCR in PC3 cells after transfection with specific (MALAT1) or control (LacZ) gapmers at 24h and 72h upon gapmers delivery. Data represented as mean of fold change vs LacZgapmer +/- SEM (N=3). * p<0.05 MALAT1gapmer vs LacZgapmer.

A

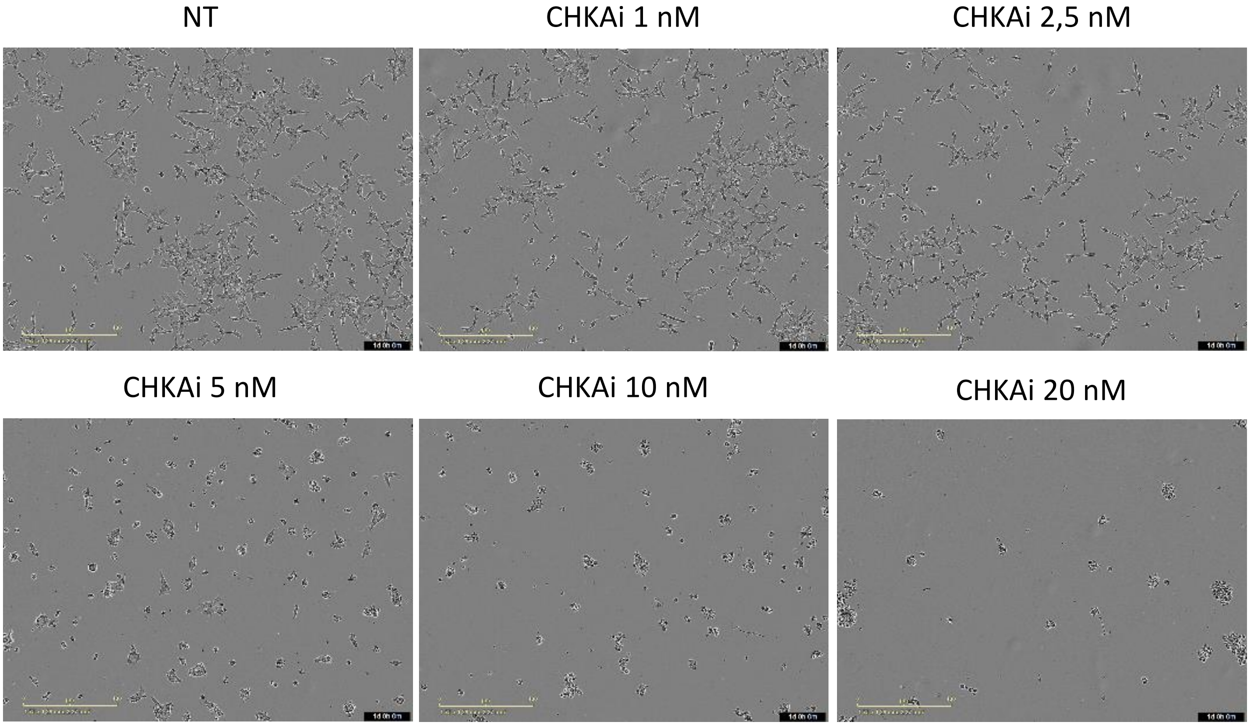

B

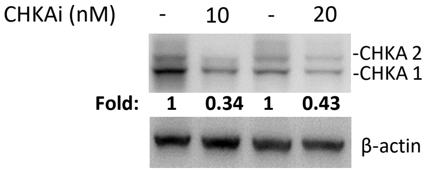

Figure S2. Effects of CHKA inhibitor treatment on cell proliferation. A) raw data pictures of cell confluence exported from the IncuCyte system after 24 h incubation (see Fig. 3A); the scale bars represent 400 µm; cell confluences after 24 h treatment with CHKA inhibitor. B) Representative CHKA western blot after24 h treatment with CHKA inhibitor. β-actin was used as loading control. Numbers represent fold change vs normalized control.

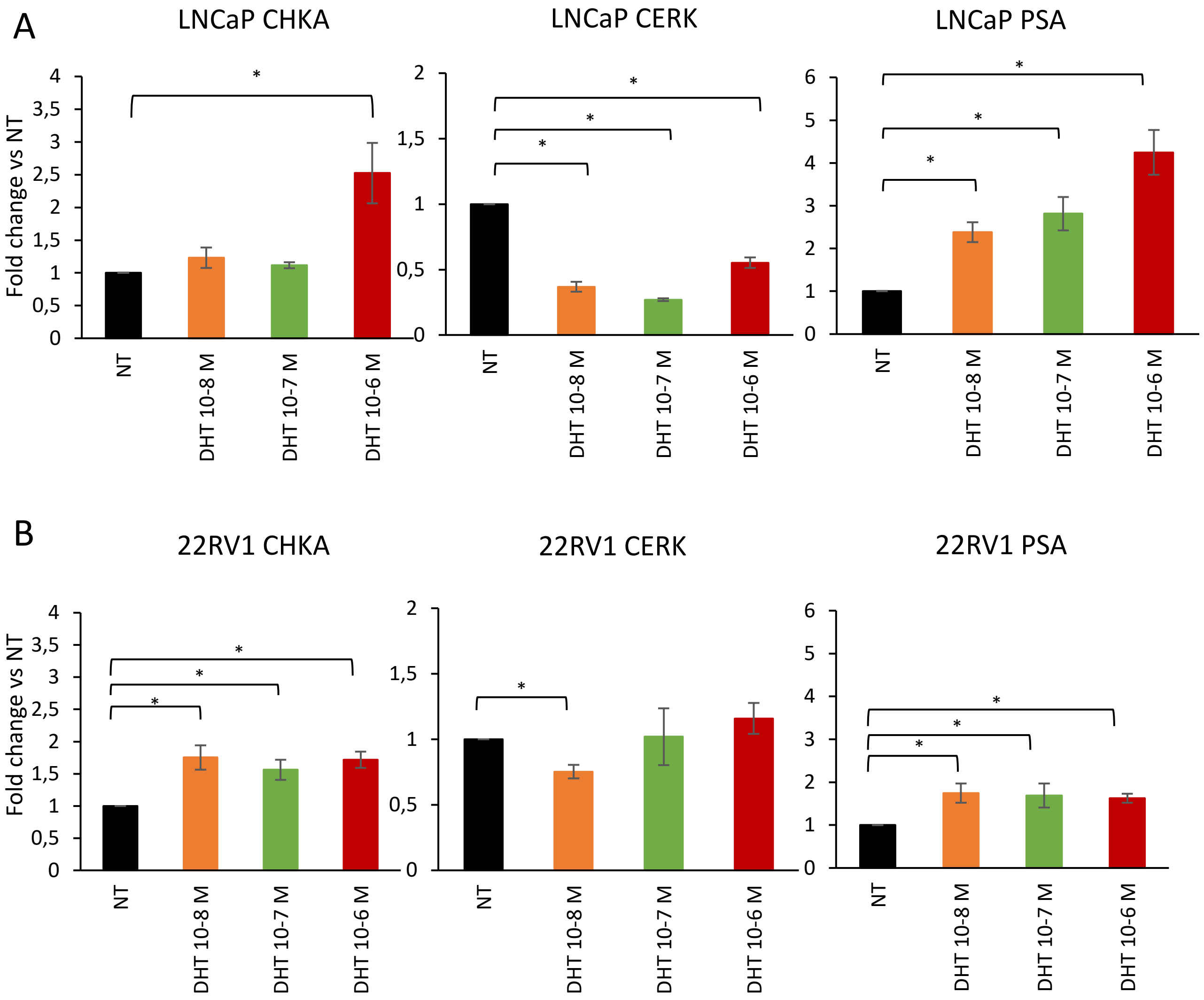

Figure S3. Effects on CHKA expression in Dihydrotestosterone (DHT) treated PCa cells. A-B) CHKA, CERK and PSA transcripts quantified by qRT-PCR in LNCaP (A) and 22RV1 (B) cell lines. Cells were treated with DHT at different concentration (10^{-8} M, 10^{-7} M, 10^{-6} M) for 16 hour. Data represented as mean of fold change vs not treated (NT) +/- SEM (N=3). * $p<0.05$ DHT vs NT.

A

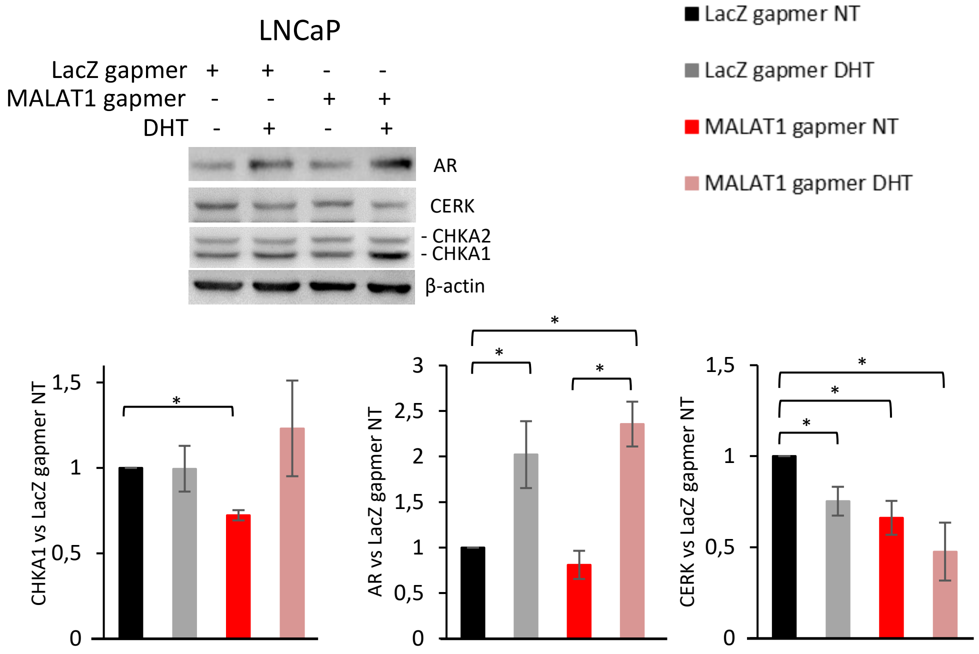

B

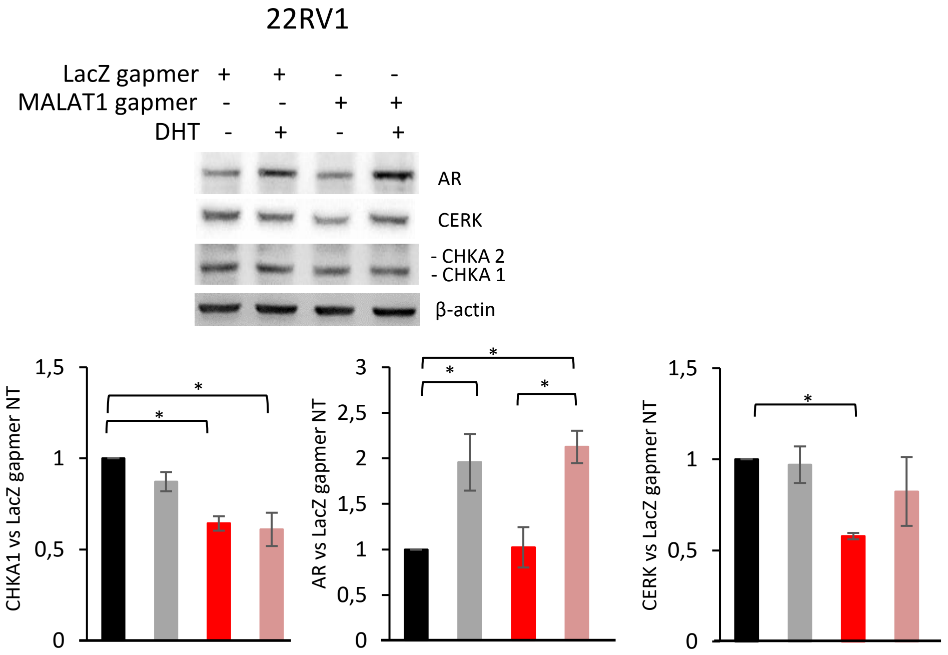

Figure S4. Effects on CHKA expression in MALAT1 depleted PCa cells in combination with Dihydrotestosterone (DHT) treatment. A-B) Representative western blot and densitometry analysis for AR, CERK and CHKA in LNCaP and 22RV1 cells after MALAT1 gapmers transfection (16h of transfection for LNCaP, A; 24h of transfection for 22RV1, B) treated with or without DHT (10^{-6} M for LNCaP and 10^{-8} M for 22RV1) for 16 hour. β-actin was used as loading control. Data represented as mean of fold change vs LacZgapmer +/- SEM (LNCaP N=3; 22RV1 N=4). * $p<0.05$.

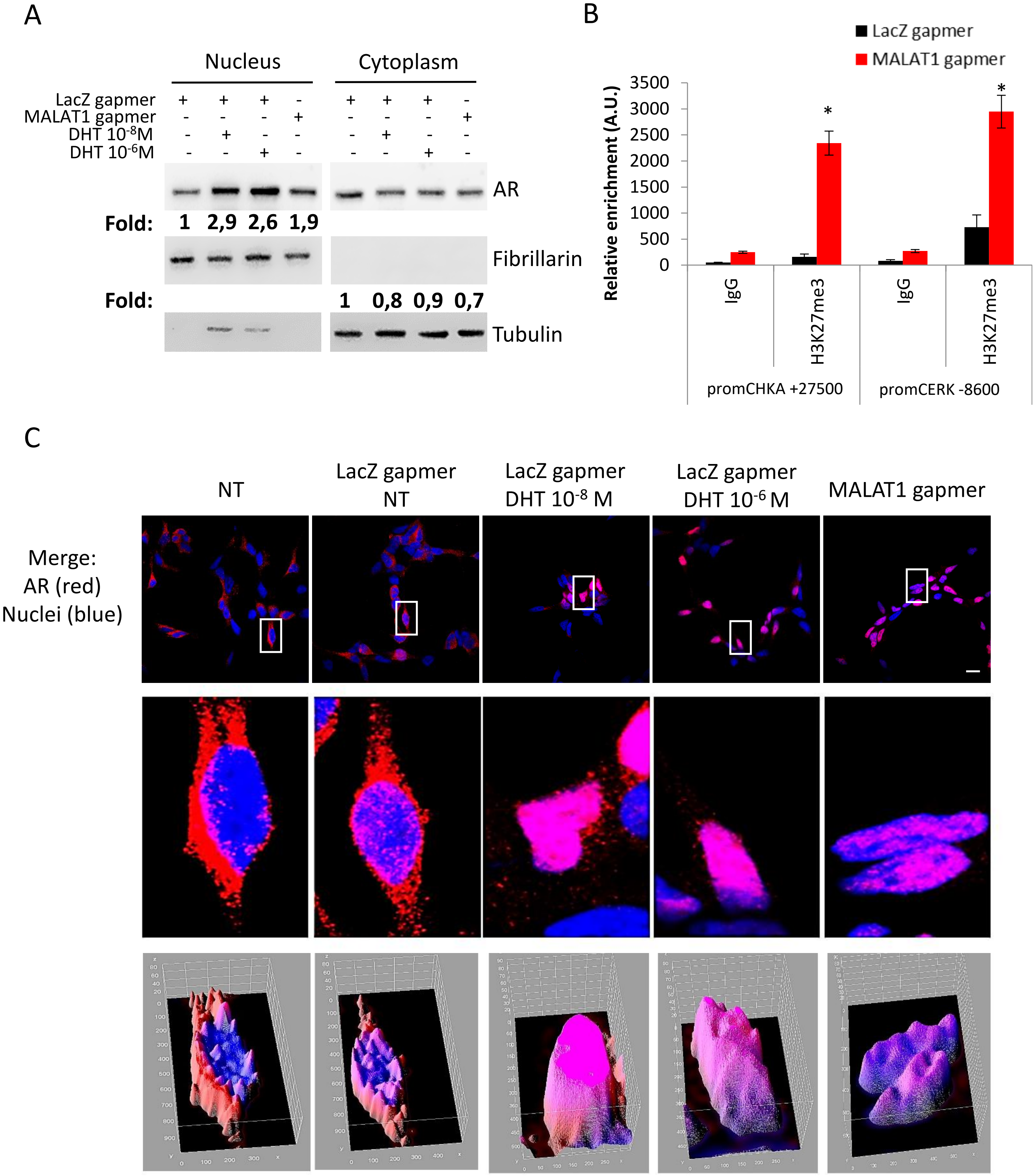

Figure S5. AR nuclear translocation and epigenetic modification upon MALAT1 targeting or dihydrotestosterone (DHT) treatment. A) Representative western blot (of two independent experiments performed) reflecting AR protein levels using fractionated cells extracts (nuclear or cytoplasmatic extracts) of LNCaP cells upon MALAT1 gapmer delivery (16h) or DHT treatment (10^{-8} M and 10^{-6} M 4h). Fibrillarin and Tubulin were used as loading control. Numbers represent fold change vs LacZ gapmer after normalization to loading control. White lines indicate extracts that were run in noncontiguous lanes in the same gel. B) H3K27me3 histone tail modification on CHKA and CERK genome regions after MALAT1 gapmers transfection (16 hours). IgG served as negative control. Data are represented as relative enrichment in Arbitrary Units (AU). *$p<0.05$ MALAT1gapmer vs. LacZgapmer. C) Selected area from confocal images (Merge in figure 7C) were digitally transformed (bottom panels) to show red fluorescence intracellular distribution, nucleus vs cytoplasm (see methods); white squares indicated zoom area in middle panel.

Table S1:Absolute quantification (nmole/10 ^{6} cells) of aqueous metabolites in PCa

Metabolic pathways	Metabolites	Cell lines			
		PC3		C27IM	
		Lacz	MALAT1	Lacz	MALAT1
Glucose metabolism	D-glucose	7.51	3.94	bd	bd
	Lactic acid	32.59±18.42	28.21±8.38	10.85	6.35
Nucleotide metabolism	AMP	4.03±0.67	2.37±1.01	1.74	1.23
	NAD	2.44±0.70	1.97±0.60	1.42	1.08
	NADP	1.15±0.75	0.80±0.57	0.18	0.29
	ADP+ATP	3.57±2.44	2.73±1.49	11.68	7.63
	UDP-glucose	6.51 ± 1.10	3.97±0.36	2.73	2.56
	UDP-glc_NAC	14.30 ± 4.20	10.78±5.61	3.65	3.63
One carbon metabolism	Formic acid	56.94±6.83	51.32±10.02	19.01	54.72
Aminoacid metabolism	L-tyrosine	5.14±1.57	2.22±0.95	2.41	2.11
	L-histidine	1.48±0.21	1.0±0.43	0.87	0.42
	L-glycine	7.90±1.43	5.22±1.32	9.52	0.88
	L- glutamic acid	38.59±5.31	29.30±1.94	46.31	25.41
	L- valine	8.41±2.62	1.61±0.05	3.46	1.92
	L- glutamine	16.84±4.34	10.42±1.33	28.31	16.01
	L -isoleucine	5.25±2.65	2.34±0.30	4.54	2.62
	L-aspartic acid	9.59±0.15	6.25±0.43	26.36	13.97
	L-alanine	11.75±5.69	5.40±0.72	7.60	4.27
Redox balance metabolism	Glutathione	28.43±1.88	21.65±0.25	36.82	20.89
	taurine	7.58±1.07	5.13±1.07	6.76	3.38
Lipid metabolism	GPC	bd	0.61±0.25	3.04	1.72
	PCho	19.60±1.75	6.95±0.51	11.86	5.58
	Cho	2.25±1.92	1.89±1.11	0.48	0.13
	Myo-inosytol	15.49±4.32	11.62±2.69	3.71	6.22
Lipid and aminoacid metabolism	Acetic acid	13.05±6.93	3.34±0.74	3.06	4.03
	3-hydroxy butyric acid	8.21±3.08	6.04±3.54	10.90	9.01
Krebs Cycle	Succinic acid	5.73±3.86	1.98±0.43	1.97	1.18
	Total creatine	15.67±0.43	10.08±1.10	15.81	8.26

Abbreviations: choline (Cho); glycerophosphocholine (GPC); Phosphocholine (PCho); Uridine diphosphate N-acetylglucosamine (UDP-glc-NAC).
